# An optimized HEK293T cell expansion protocol using a hollow-fiber bioreactor system

**DOI:** 10.1093/biomethods/bpad018

**Published:** 2023-09-13

**Authors:** Nathan D Frank, Mindy Miller, Dalip Sethi

**Affiliations:** Research and Development, Terumo BCT, Lakewood, CO, United States; Research and Development, Terumo BCT, Lakewood, CO, United States; Research and Development, Terumo BCT, Lakewood, CO, United States

**Keywords:** automated, cell culture, HEK293T, hollow fiber bioreactor, quantum

## Abstract

Viral vectors are commonly used to introduce genetic material into cells to modify cell function for a variety of purposes. Manufacture of those modified viruses may use a variety of cell types to generate high titers of viral particles; one of the most common being HEK293 cells. These cells have been modified into different lines aimed at satisfying specific use cases. HEK293T cells, for example, have been modified to include the SV40 large T antigen. Efficient viral particle production by HEK293T cells requires the maintenance of favorable cell culture conditions during expansion and transfection. This protocol describes the use of the Quantum^®^ hollow-fiber bioreactor (HFB) system for the automated expansion of HEK293T cells, and the results derived using the protocol described herein were not compared with those from tissue culture flasks or other expansion platforms, as the parameters described are unique to Quantum’s hollow fiber cell expansion environment. The purpose of this protocol is to help users of Quantum to focus on relevant parameters of expansion in the HFB milieu and to provide guidelines for a successful expansion of HEK293T cells in the Quantum system. The steps provided have been optimized to reliably control environmental factors related to glucose, lactate, and pH. Data reflecting this consistency are provided along with procedural time points reflected in text and figure formats.

## Background

Precision-engineered viruses/viral vectors are a useful tool used to deliver genetic material for altering cell function to research and cure disease. Lentiviruses, adenoviruses, and adeno-associated viruses are uniquely equipped for this task, and having multiple strain options available makes viral transduction a versatile technique that allows researchers to consider the advantages and disadvantages of each strain. This, in turn, has made viral vectors applicable to a wide range of fields, such as hematology [1], oncology [2], neurology [2], pulmonology [3], hepatology [4], urology [5], rheumatology [6], and ophthalmology [7], with clinical trials and animal-based research showing improvements for several conditions, such as hemophilia, AIDS, glioblastoma, melanoma, prostate cancer, breast cancer, pancreatic cancer, kidney cancer, hepatocellular carcinoma, ovarian cancer, Parkinson’s disease, Alzheimer’s disease, Rett Syndrome, Huntington’s disease, cystic fibrosis [2], rheumatoid arthritis [6], and macular degeneration [7].

HEK293 cells have been used to investigate signal transduction, develop vaccines, test drugs, study protein interactions, produce recombinant proteins, and investigate the effects of environmental toxins and are among the most common cell lines used in generating viral vectors. A range of modified HEK293 cells have been generated to serve more specific functions. Among these modified cells are HEK293T cells designed to produce high titers of viral vectors for cell and gene therapies as they have been modified to include the SV40 large T antigen to increase the level of protein expression when the cells are transfected [8, 9]. Indeed, previous studies showed a statistically significant difference in which HEK293T cells produced a viral titer that was approximately 10 times that produced by HEK293 cells [10]. They also showed that HEK293T cells could undergo more passages without affecting viral production compared to HEK293 cells [10].

The efficiency of cell expansion and viral vector production by HEK293T cells is highly dependent on the methods and cell culture modality that are used for expansion and transfection. Several cell culture modalities have been explored for this purpose, including fixed-bed bioreactors, roller bottles, stirred tank reactors, 2D cell factories, standard T-flasks, and hollow-fiber bioreactors (HFBs) [11]. This protocol describes HEK293T expansion in the HFB of the Quantum^®^ system (Terumo BCT, Lakewood, CO, USA). Maintaining a consistent cell culture environment can prove difficult when using manual techniques. In flasks, lactate levels tend to spike prior to a change of medium and drop after a fresh medium is introduced to the system, while glucose concentration displays the opposite trend. Moreover, the oxygen demands of an expanding cell population may exceed the diffusive capacity of a static volume of medium, resulting in less-than-optimal growth [12].

In addition to environmental consistency, another challenge is the semi-adherent nature of HEK293T cells, which have been known to detach from their culture surface during expansion if handled improperly. While this can be managed through careful technique during manual expansion, automated expansion in an HFB requires a unique protocol based on precise management of the fluid flow of medium or buffer through the system. By decreasing the circulation rate of intracapillary (IC) media, the cells were better retained in the bioreactor with greatly reduced loss to the waste line. This concept was also applied to the cell washing process that occurs in the fibers of the HFB prior to the addition of cell disassociation reagent to ensure that cell loss to the washout was reduced or eliminated.

To address the issues with manual cell culture, Quantum has been designed as an automated, functionally closed device to manage the subtle fluidic manipulations required to seed, maintain, and harvest various adherent and suspension cell types for the manufacture of cell and gene therapies. Quantum has been used to culture mesenchymal stem cells for regenerative medicine applications [13, 14], neural stem cells for recurrent brain tumors [15], and T cells for immunotherapies [16] and has been demonstrated to be a cost-effective alternative to other culture modalities for the scale-up and scale-out of cell expansion [17, 18].

The hollow fibers of the Quantum HFB have IC and extracapillary (EC) sides separated from one another by a porous, 50-µm thick membrane through which the two sides can communicate. One unique feature of Quantum is that cells are cultured on the IC side of the membrane, while fluid on the EC side is circulated through the gas transfer module integrated into Quantum’s disposable set making use of perfusion to provide a consistent exchange of oxygen, nutrients, and waste to facilitate cell expansion. Standard adherent cell protocols on Quantum can utilize relatively high inlet and circulation rates as fresh media flows unidirectionally through the fibers and thus across the cells. The pores in the fibers are large enough to allow the diffusion of small molecules, such as gases, salts, lactate, and glucose, through the membrane but small enough to retain cells and larger proteins in the IC lumen.

The use of an IC side for cell culture presents advantages over previous approaches to culturing virus-producing cell lines. Past attempts to culture virus-producing cell lines in non-Quantum HFBs maintained the cells on the EC side rather than on the IC side as in Quantum [19]. This adds a range of complicating factors to the expansion process relative to Quantum culture. When the cells are cultured in this fashion, harvesting the cells will be difficult due to the nonlinear nature of fluid movement on the EC side of the bioreactor and, due to the difficulty of effectively harvesting cells, cell numbers must be estimated from T-flask confluency counts rather than counting directly. Estimating rather than counting the cells can lead to improper multiplicities of infection when cells are exposed to viruses. The convoluted structure of the EC side of an HFB can also lead to the establishment of differential microenvironments for cell expansion. These issues are alleviated in cell expansion performed in Quantum as the IC side of the fibers is linear and cells can be easily harvested with high efficiency.

This protocol is designed to optimize the expansion and harvest of HEK293T cells when using Quantum as measured by cell number and the condition of the surrounding media. To note, that the cell harvest yield or doubling times of cultured cells using the protocol described here is not intended to be compared to that in tissue culture flasks or any other expansion platform beyond Quantum as the parameters described are unique to Quantum’s hollow fiber cell expansion environment. The purpose of this protocol is to help users of Quantum to focus on relevant parameters of expansion in Quantum and to provide guidelines for a successful expansion of HEK293T cells in the system. This protocol was specifically designed for users’ convenience. As the Quantum expansion presented herein takes roughly 100 h or approximately 4 days, the bioreactor can be set up on a Thursday or Friday so that cells are ready for harvest or transfection the following Monday or Tuesday, respectively. Moreover, the seeding numbers used were chosen to produce about 2 billion cells at harvest. The number of cells seeded and the duration of the culture can be modified for each specific application.

Importantly, this protocol relies on maintaining the proper environment for cells to expand. Two of the most important parameters for proper growth are pH and pre-seeding cell health. Prior experiments showed that pH had dropped below 7.2 when lack of adherence and cell accumulation in the waste bag was observed. Thus, the protocol was designed to include EC inlet rates capable of maintaining pH at or above 7.3 without altering IC parameters. Successful use of non-pH-altering generation of lentivirus has been demonstrated in Quantum [20]. Furthermore, different pre-seeding culture methods may result in different growth kinetics. In our experience, conditions were optimal when cells were taken directly from cryostorage and kept in the log phase during pre-expansion.

## Materials

### Reagents

The following materials were used.

- Phosphate-buffered saline (PBS) (Lonza, Walkersville, MD, USA, Cat. #95042-490).
- Vitronectin (VN) Recombinant Human Protein (10 ml) (ThermoFisher, Waltham, MA, USA, Cat. # A31804).
- Fetal bovine serum (FBS), USDA approved Origin (Heat Inactivated) (Corning, Glendale, AZ, USA, Cat. #35-011-CV).
- Dulbecco’s Modified Eagle Medium (DMEM), high glucose, GlutaMAX™ Supplement (ThermoFisher, Waltham, MA, USA, Cat. #10569044).
- Penicillin–Streptomycin–Neomycin (PSN) antibiotic mixture (ThermoFisher, Waltham, MA, USA, Cat. #15640055).
- 0.25% Trypsin–EDTA (ThermoFisher, Waltham, MA, USA, Cat. #25200114).

### Recipes

- VN coating solution: mix 5 mg of VN in 10 ml of thawed solution with 90 ml of PBS.
- Base media: DMEM only.
- Complete media: DMEM + 10% FBS + 1× PSN antibiotic

### Equipment

- Centrifuge.
- Serological motorized pipette controller.
- Sterile tubing welder (TSCD-Q, TerumoBCT).

### Disposables

- T175 flasks.
- Five-stack layer flasks.
- Quantum cell expansion set (Cat# 21037).
- Cell inlet bags (Cat# 21035).
- Media bags (Cat# 21026).
- Waste bags (Cat# 21027).
- TSCD-Q wafers (Cat# SC*W017).
- Serological pipettes.

## Procedure

**Figure 1:**
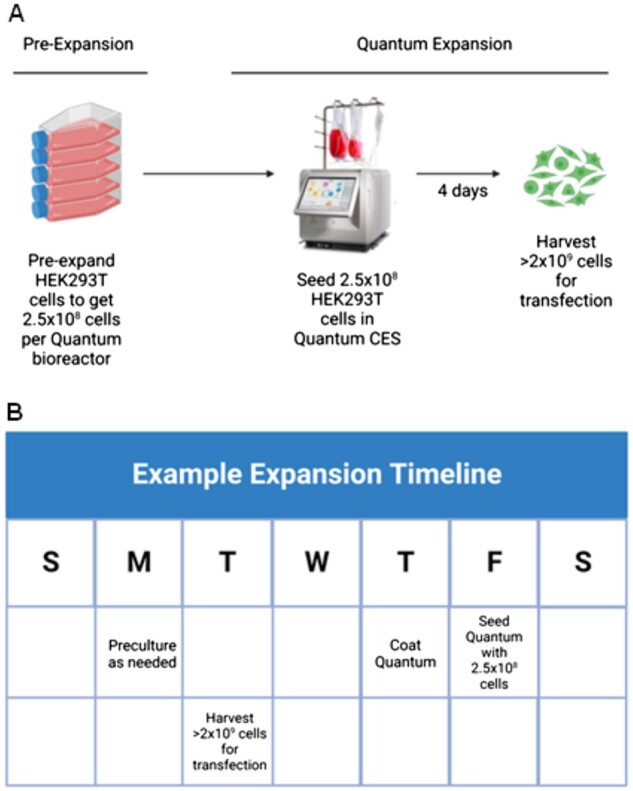
(**A**) The main steps are obtaining enough cells to begin expansion followed by seeding, expanding, and harvesting. (**B**) A sample schedule for seeding.

A schematic of the major phases of the protocol is shown in Fig. 1.

1. Pre-expansion (Note: Pre-expansion may use any method to generate at least 250 million cells per Quantum. Below is an example method utilizing flasks that can be used to generate enough cells to load three Quantum Cell Expansion systems but pre-expansions may also be carried out in Quantum).

1.1 Thaw a sufficient number of cells to recover 8.5 × 10^7^ viable cells.

1.2 Seed five T175 flasks at ∼1.7 × 10^7^ viable cells/flask in 50 ml complete media per flask and expand for 96 h or until culture reaches ∼90% confluency.

1.3 Harvest flasks by washing cells two times with PBS and then incubating with 20 ml trypsin per flask for 8 min.

1.4 Dilute trypsinized cells at a ratio of 1:2 with complete media, pool samples by transferring to a single 500 ml conical, and centrifuge for 5 min at 500*g*.

1.5 Resuspend the combined cells in 500 ml complete media and pass them into one 5-stack layer flask and culture for 72 h or until culture reaches ∼90% confluency.

1.6 Split cells evenly three ways, or count and use 2.5 × 10^8^ cells per Quantum run and fill a cell inlet bag with cells using a 50 ml syringe.

**Figure 2:**
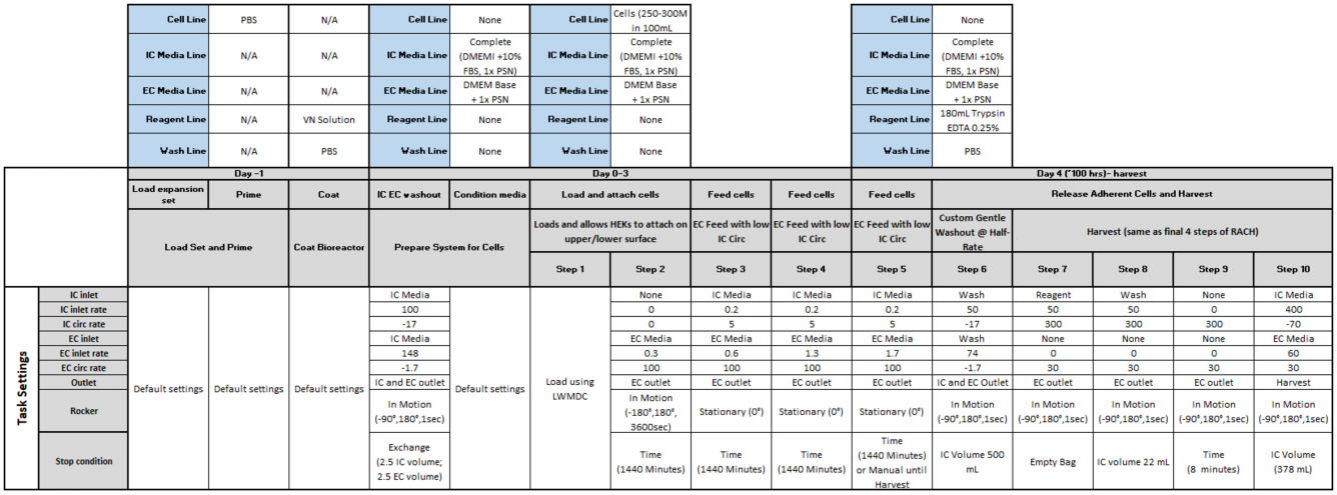
Detailed steps for programming the expansion process.

2. Quantum expansion (Note: Please refer Fig. 2 for complete details on media usage and task settings).

2.1 On the day prior to loading cells, prime the system, prepare VN, coat the bioreactor, and fill media bags:

2.1.1 Filter 5 l PBS into a 5 l media bag per Quantum run (Note: PBS filtration maybe performed several days in advance).

2.1.2 Attach a Quantum disposable set and prime with PBS using the integrated prime cell expansion set task.

2.1.3 Prepare the VN solution in a biosafety cabinet. The recipe below will coat one Quantum.

2.1.3.1 Allow one 5 mg vial of VN to thaw at room temperature.

2.1.3.2 Add 90 ml of PBS to the top of an appropriately sized vacuum/filter storage system followed by 10 ml VN solution.

2.1.3.3 Filter VN solution using a vacuum pump.

2.1.3.4 Fill the cell inlet bag with filtered VN solution using a 50 ml syringe.

2.1.4 Connect the VN solution bag to the reagent line of the Quantum^®^ system using a sterile welder.

2.1.5 Use the integrated coat bioreactor task to coat each bioreactor overnight.

2.1.6 Prepare a base media bag containing 5 l total of base media.

2.1.7 Prepare a complete media bag containing 5 l of complete media.

2.1.8 Sterile weld the base media bag to the EC inlet line.

2.1.9 Sterile weld the complete media bag to the IC inlet line.

2.1.10 On the day following the coating, initiate the IC/EC washout task using IC media as the inlet source for both IC and EC sides.

2.1.11 Once the washout is complete, initiate the condition media task.

2.1.11.1 Once the system reaches Step 2 of the condition media task, draw a baseline media sample from the EC sample port using a luer lock syringe. This will provide a baseline reading for glucose and lactate values to compare future readings throughout the expansion.

2.2 Load and attach cells.

2.2.1 Attach the cell inlet bag containing 2.50 × 10^8^ HEK293T cells to the cell inlet line using a sterile welder.

2.2.2 Load into the system using the “load with multiple distribution cycles (LWMDC)” task.

2.2.3 Attach cells overnight using a custom task with parameters defined in Fig. 2 (Note: The bioreactor moves between −180° and 180° every 3600 s during cell attachment and is stationary during feeding).

2.3 Feed schedule

2.3.1 Feed cells for 3 days following attachment using EC inlet to feed with low IC circulation.

2.3.2 EC circulation rates are maintained at 100 ml/min, and EC inlet rates are increased every 24 h.

2.3.2.1 EC inlet rate on Day 1 is 0.6 ml/min.

2.3.2.2 EC inlet rate on Day 2 is 1.0 ml/min.

2.3.2.3 EC inlet rate on Day 3 is 1.3 ml/min.

2.3.2.4 EC inlet rate on Day 4 (morning of harvest) is 1.7 ml/min with EC.

2.3.3 Keep IC feed rates at 0.2 ml/min and IC circulation rates at 5 ml/min.

Caution: High IC rates may increase shear stress, thus resulting in reduced cell adherence and increased accumulation of cells in the waste bag.

Note: EC inlet rates may be adjusted to account for glucose supply, lactate levels, and pH.

2.4 Harvest at ∼100 h (afternoon on Day 4).

2.4.1 Modify the release of adherent cells and harvest task.

2.4.1.1 Modify Step 1 (washout) to halve the IC inlet rate from 100 to 50 ml/min and the EC inlet rate from 148 to 74 ml/min. Also, change the stop condition to an IC Volume of 500 ml.

2.4.1.2 Modify Step 4 (incubate) from a 4-min incubation with trypsin to an 8-min incubation (Note: The selected washout parameters were intended to keep cells attached to the bioreactor membrane by minimizing flow rates and shear stress while still providing a full washout of the system).

2.4.2 Fill a cell inlet bag with 180 ml of Trypsin in a biosafety cabinet and sterile weld the bag to the reagent line.

2.4.3 Initiate the modified release adherent cells and harvest task.

2.4.4 Remove and weigh the harvest bag to determine harvest volume.

2.4.4.1 Subtract the weight of an empty harvest bag from the total weight.

2.4.5 Wash and count cells for downstream application.

## Anticipated results

**Figure 3:**
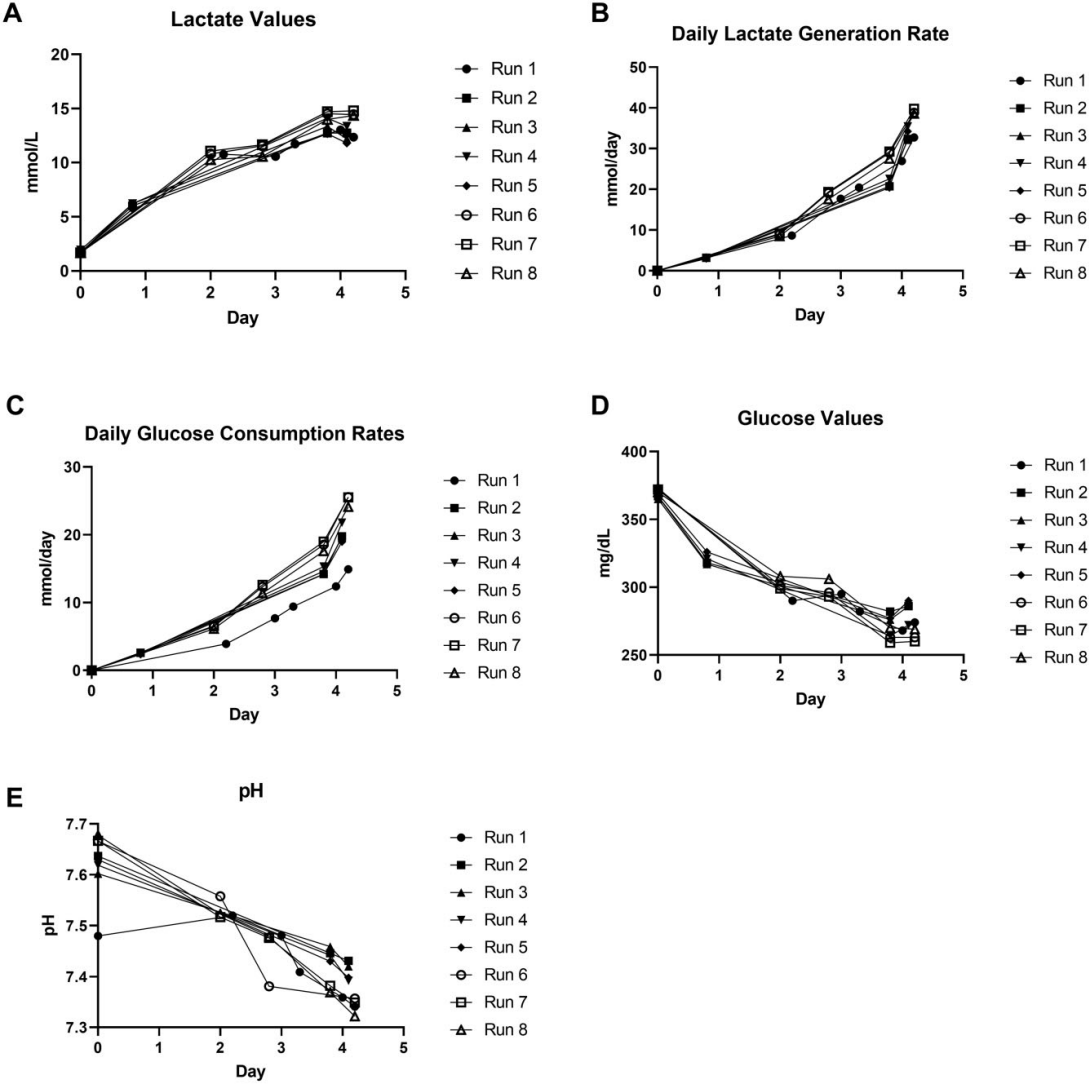
Effects of expansion on metabolism over 100 h. (**A**) Lactate levels increased over 100 h. (**B**) Lactate generation rate increased over 100 h. (**C**) Glucose consumption rate increased over 100 h. (**D**) Glucose levels decreased over 100 h. (**E**) The pH decreased over 100 h but was maintained above 7.3.

Data from three separate runs showed an average of 2.14 × 10^9^±0.18 × 10^9^ (range 2.00 × 10^9^–2.34 × 10^9^) viable HEK293T cells expanded from a seed of 2.50 × 10^8^ viable cells (viability of the cell population seeded into Quantum from pooled flasks was >98% for all runs), with an average seeding efficiency of 90%±0.4% (range 89–90%). This translates from 9.5 × 10^4^ to 1.1 × 10^5^ cells/cm^2^. The viability of the harvested cell populations was >96% for all Quantum runs described. Figure 3 shows the effects of expansion on metabolism over 100 h. Lactate levels increased by similar amounts across eight runs (within 5 mmol/l of each other). The rate at which lactate was generated was also similar across runs, as all runs were within 10 mmol/day of each other. Glucose consumption rates increased over 100 h with a corresponding decrease in glucose levels. Finally, protocol parameters enabled pH to remain above 7.3 throughout the procedure.

## Troubleshooting

1. Loss of cells to waste:
  - This protocol was designed using relatively gentle circulation rates in the bioreactor to limit the loss of detaching cells to the waste line. If cell aggregates are visible in the waste line or the outlet bag, the following might be used to address the issue.
    - High-confluency cell population: Different lines of HEK293T cells may expand differently in the HFB environment. In the expansions conducted for this work, lactate values were typically above 10.0 mmol/l by Day 2 and were above 14.0 mmol/l by Day 4. Lactate values that are notably higher than this range may indicate a higher-than-expected number of cells in the bioreactor and may require harvesting earlier than anticipated to avoid loss to the waste line.
    - Loosely attached cells: Different lines of HEK293T cells may adhere differently to the coated membrane of the HFB. If lactate and glucose readings fall withing typical ranges for the culture period, cells may be detaching even under the mild shear forces created in the HFB.
      - Reducing the IC circulation rate from the recommended speed may help to retain cells attached to the fibers. Although not tested in this study, IC circulation rates might reasonably be reduced from 5 to 2–3 ml/min to help retain cells in the system.
      - Ensure that the “Coat Bioreactor” task proceeds for 16 hours or more. Less than this amount of time may result in incomplete coating of the fiber membrane
2. Lower-than-expected harvest yield
  - If typical lactate values are achieved, but the number of cells harvested from the system is notably lower than expected, then a second harvest event can show if unharvested cells are left in the system. This procedure will require attaching a second bag to the Harvest line and the preparation of a second 180 ml bag of disassociation enzyme solution to the Reagent line. The “Release Adherent Cells and Harvest” task will need to be rerun. Make sure that an additional 1.6 l of PBS is available for the IC/EC washout step.
3. pH too low
  - This may be a result of a population of cells expanding faster than the volume of media introduced to the system can accommodate. If pH is lower than ∼7.3 for an extended period, cells may begin to detach. Introducing more medium per unit of time can help to keep the pH of the system within the desirable range. The EC inlet pump should be used for this purpose, not the IC inlet. Increasing the EC inlet rate by 10% increments relative to recommended values until the pH stabilizes within the desired range is a reasonable strategy to approach this issue. This will help to bring the pH back into line with expectations while minimizing media use for the expansion process.
4. Aggregated product
  - HEK293T cells may aggregate if they become overconfluent on the culture surface. Typically, the trituration of the HEK293T cell solution is sufficient to break aggregates down into a single-cell suspension. If aggregates persist after repeated pipetting up and down, exposure to a lower concentration of trypsin (e.g. 0.05%) may help to attain a single-cell suspension.

## Authors’ contributions

Nathan D. Frank (Conceptualization [equal], Formal analysis [lead], Investigation [lead], Methodology [lead], Writing—original draft [lead]), Mindy Miller (Conceptualization [equal], Investigation [equal], Methodology [equal], Writing—original draft [equal], Writing—review & editing [equal]), and Dalip Sethi (Conceptualization [equal], Funding acquisition [equal], Project administration [lead], Supervision [equal], Writing—original draft [supporting], Writing—review & editing [equal])

*Conflict of interest statement*. All authors are employees of Terumo Blood and Cell Technologies, Inc. (Lakewood, CO, USA).
